# Targeting Nrf2-Mediated Oxidative Stress Response Signaling Pathways as New Therapeutic Strategy for Pituitary Adenomas

**DOI:** 10.3389/fphar.2021.565748

**Published:** 2021-03-24

**Authors:** Xianquan Zhan, Jiajia Li, Tian Zhou

**Affiliations:** ^1^ Shandong Key Laboratory of Radiation Oncology, Cancer Hospital of Shandong First Medical University, Jinan, China; ^2^ Science and Technology Innovation Center, Shandong First Medical University, Jinan, China; ^3^ Department of Oncology, Shandong Provincial Hospital Affiliated to Shandong First Medical University, Jinan, China; ^4^ Department of Thoracic Surgery, Xiangya Hospital, Central South University, Changsha, China

**Keywords:** pituitary adenoma, oxidative stress, Nrf2, signaling pathway, biomarker, therapeutic target and drug

## Abstract

Oxidative stress and oxidative damage are the common pathophysiological characteristics in pituitary adenomas (PAs), which have been confirmed with many omics studies in PA tissues and cell/animal experimental studies. Nuclear factor erythroid 2 *p*45-related factor 2 (Nrf2), the core of oxidative stress response, is an oxidative stress sensor. Nrf2 is synthesized and regulated by multiple factors, including Keap1, ERK1/2, ERK5, JNK1/2, *p*38 MAPK, PKC, PI3K/AKT, and ER stress, in the cytoplasm. Under the oxidative stress status, Nrf2 quickly translocates from cytoplasm into the nucleus and binds to antioxidant response element /electrophile responsive element to initiate the expressions of antioxidant genes, phases I and II metabolizing enzymes, phase III detoxifying genes, chaperone/stress response genes, and ubiquitination/proteasomal degradation proteins. Many Nrf2 or Keap1 inhibitors have been reported as potential anticancer agents for different cancers. However, Nrf2 inhibitors have not been studied as potential anticancer agents for PAs. We recommend the emphasis on in-depth studies of Nrf2 signaling and potential therapeutic agents targeting Nrf2 signaling pathways as new therapeutic strategies for PAs. Also, the use of Nrf2 inhibitors targeting Nrf2 signaling in combination with ERK inhibitors plus p38 activators or JNK activators targeting MAPK signaling pathways, or drugs targeting mitochondrial dysfunction pathway might produce better anti-tumor effects on PAs. This perspective article reviews the advances in oxidative stress and Nrf2-mediated oxidative stress response signaling pathways in pituitary tumorigenesis, and the potential of targeting Nrf2 signaling pathways as a new therapeutic strategy for PAs.

## Introduction

Pituitary adenoma (PA) is a common intracranial neoplasm that occurs in the central regulatory organ pituitary gland in the hypothalamic-pituitary-target organ axis system, which seriously affects human endocrine system and health. PAs account for 10–25% of all intracranial tumors, and are classified into benign (∼65%), invasive (∼35%), and malignant (carcinoma; only 0.1–0.2%) PAs according to the malignancy level (Stalla et al., 2019). PAs are divided into macroadenomas (≥10 mm) and microadenomas (<10 mm) according to tumor size (Lopes, 2017). They are also divided into clinically functional and nonfunctional PAs (FPAs and NFPAs) according to the level of hormone secretion (Zhan et al., 2016). FPAs are hormone-secreting PAs, which result in hyperpituitarism, including acromegaly derived from growth hormone (GH)-secreting PAs, hyperprolactinemia derived from prolactin (PRL)-secreting PAs, and Cushing’s syndrome derived from adrenocorticotropin (ACTH)-secreting PAs. NFPAs are non-hormone-secreting PAs (Qian et al., 2018). The main clinical symptoms of PAs include inappropriate hormone secretion syndrome, and compression of the neighboring tissues and structures such as headache, visual field defect, and increased intracranial pressure (Reimondo et al., 2019). PA is a multi-factor, multi-process, and multi-consequence complex disease, which is involved in a series of molecular alterations at the levels of genome, transcriptome, proteome, peptidome, metabolome, and radiome; and these molecules mutually associate and function in a molecular network system (Zhan and Desiderio, 2010b; Hu et al., 2013; Grech et al., 2015; Cheng and Zhan, 2017; Lu and Zhan, 2018). Thus, one must shift the research and practice strategy from a single-factor model to a multi-parameter systematic model for predictive, preventive, and personalized medicine in PAs (Hu et al., 2013; Grech et al., 2015; Cheng and Zhan, 2017). Multiomics is an effective approach to realize this multi-parameter systematic strategy model shift, which can establish signaling pathway systems for in-depth understanding of molecular mechanisms of PAs, identify molecular network-based biomarkers for prediction, diagnosis, and prognostic assessment of PAs, and discover signaling pathway network-based therapeutic targets for effective treatment of PAs (Grech et al., 2015; Cheng and Zhan, 2017; Lu and Zhan, 2018).

A series of omics analyses have been performed in PAs to reach our long-term goals that clarify molecular mechanisms and discover effective biomarkers and therapeutic targets for PAs (Zhan and Desiderio, 2010a; Long et al., 2019; Cheng et al., 2019; Wang Y. et al., 2019), including NFPA quantitative transcriptomics (differentially expressed genes, DEGs) (Moreno et al., 2005; Cheng et al., 2019), NFPA quantitative proteomics (differentially expressed proteins, DEPs) (Moreno et al., 2005), NFPA proteomic mapping (Zhan and Desiderio, 2003; Wang X. et al., 2015; Cheng et al., 2019), NFPA nitroproteomics (Zhan and Desiderio, 2006), invasive NFPA quantitative transcriptomics (Galland et al., 2010; Zhou et al., 2011; Wang Y. et al., 2019), invasive NFPA quantitative proteomics (Zhan et al., 2014b), control pituitary proteomic mapping (Beranova-Giorgianni et al., 2002; Giorgianni et al., 2003; Zhao et al., 2005), pituitary control nitroproteomics (Zhan and Desiderio, 2004; Zhan and Desiderio, 2007), control pituitary phosphoproteomics (Giorgianni et al., 2004; Beranova-Giorgianni et al., 2006), PRL-secreting adenoma proteomics and transcriptomics (Evans et al., 2008), and ACTH-secreting adenoma proteomics and metabolomics (Feng et al., 2018). Integrative analysis of these omics data has revealed some important signaling pathway network alterations in PA pathogenesis, including mitochondrial dysfunction, oxidative stress, cell cycle dysregulation, and mitogen-activated protein kinase (MAPK) signaling pathway alteration (Zhan and Desiderio, 2010a; Long et al., 2019). Mitochondrial dysfunction pathway network and mitochondrial dynamics (Li and Zhan, 2019), and MAPK signaling pathway-based drug therapeutic targets (Lu et al., 2019) have been discussed in detailed in PAs. It is well-known that mitochondria are the energy factories of the body, and mitochondrial metabolism is the source of reactive oxygen species (ROS). The imbalance between free radicals reactive oxygen/nitrogen species (ROS/RNS) and antioxidant system leads to oxidative stress, which plays an important role in diseases. Many studies focus on oxidative stress system as therapeutic strategy; for example, benfotiamine is an efficient antioxidant, which could prevent oxidative stress in the anterior tibialis muscle and heart of mice (Gonçalves et al., 2019). Another research shows that pancreatic oxidative damage in the diabetic state is caused by ROS, and scavenging the various ROS generated in the disease is one of effective ways to treat this disease (Afolabi et al., 2018). Studies have clearly demonstrated that mitochondrial dysfunction and oxidative stress pathway changes operate in PAs (Zhan and Desiderio, 2010a), and nuclear factor erythroid 2 p45-related factor 2 (Nrf2)-mediated oxidative stress response significantly impacts the pathogenesis of PAs and modulates the energy metabolism reprogramming for PAs (Sabatino et al., 2018). It is well-known that PAs can lead to abnormal hormone secretion, which might affect oxidative stress and Nrf2 signaling in PAs; for example, human growth hormone (hGH) can attenuate inflammation and oxidative stress attained by Cisplatin probably through inhibition of Nrf2/heme oxygenase 1 (HO-1) pathway (Mahran, 2020). More studies show that Nrf2 signaling and oxidative stress can be regulated by cortisol (Wu et al., 2019), thyroid hormone (Mishra et al., 2019), follicle-stimulating hormone (FSH) (Li et al., 2020), luteinizing hormone (LH) (Li et al., 2020), GH (Mahran, 2020), ACTH (Benlloch et al., 2016), and PRL (Ebokaiwe et al., 2020). These findings clearly demonstrate the importance of oxidative stress in PAs. This present review article will focus on oxidative stress response signing pathway network in PA pathogenesis.

## Redox Homeostasis and Nrf2 as the Heart of Oxidative Stress Response

Oxidative stress is derived from the imbalance between the upload of free radicals ROS/RNS from *in vivo* and *in vitro* environmental approaches and the ability of endogenous antioxidants to detoxify these ROS/RNS (Prasad et al., 2016; Klaunig, 2018; Sajadimajd and Khazaei, 2018). It results in the injuries of multiple biomacromolecules such as DNAs, RNAs, proteins, and membrane lipids to significantly associate with a wide spectrum of diseases including cancers. Many studies demonstrate that the increased ROS/RNS productions promote carcinogenesis development (Kudryavtseva et al., 2016; Kruk and Aboul-Enein, 2017), and oxidative stress-medicated chronic inflammation is the risk factor of tumorigenesis (Reuter et al., 2010; Qian et al., 2019). The oxidative phosphorylation system in mitochondrial respiratory chain is the central machine that generates ROS products such as superoxide radical (O_2_^.-^). One study shows that ROS levels and signs of oxidative damage are significantly increased in PAs (Sabatino et al., 2018). One of the most important RNS, nitric oxide (NO), is generated by inducible nitric synthase (iNOS) in many pathogenesis conditions, which can rapidly react with superoxide radical (O_2_^.-^) to generate more toxic peroxynitrite anion (ONOO^-^) and highly reactive hydroxyl radical (OH^.^) to attack DNAs, RNAs, proteins, and membrane lipids. iNOS has been extensively found in rat and human pituitaries (Ceccatelli et al., 1993; Lloyd et al., 1995; Ueta et al., 1998; Kruse et al., 2002; Pawlikowshi et al., 2003) and has the elevated activities in PAs compared to those in controls (Vankelecom et al., 1997; Kruse et al., 2002). Another study shows that NO functions in the hypothalamic-pituitary-adrenocortical axis (Riedel, 2002) by promoting the release of follicle-stimulating hormone-releasing hormone (FSHRH) and luteinizing hormone-releasing hormone (LHRH) from hypothalamus (McCann et al., 2001; Pinilla et al., 2001; McCann et al., 2003), and regulating secretion of PRL (Duvilanski et al., 1995) and GH in pituitaries and PAs (Cuttica et al., 1997; Pinilla et al., 1999; Bocca et al., 2000). Peroxynitrite anion (ONOO^−^) is a key factor *in vivo* that causes protein tyrosine nitration and alters protein functions. Nine nitrotyrosine-containing proteins have been identified in NFPA tissues, and tyrosine nitration occurs in important structural and functional domains to change protein functions (Zhan and Desiderio, 2006).

With the generation of ROS/RNS, the *in vivo* antioxidant detoxification system is correspondingly initiated to adapt against the increased ROS/RNS (Valko et al., 2006; Obrador et al., 2019). The endogenous antioxidant detoxification system is a very complex system, including i) enzymatic antioxidants such as superoxide dismutases (CuZnSOD and MnSOD), glutathione peroxidase, and catalase; ii) non-enzymatic antioxidants such as vitamin E, vitamin C, carotenoid, flavonoid, selenium, thiol antioxidant (thioredoxin, lipoic acid, and glutathione), and others; and iii) multiple regulatory factors [Nrf2, NF-kB (nuclear factor kB), and AP-1 (activator protein-1), etc.] that interact with antioxidants (Valko et al., 2006; Obrador et al., 2019). CuZnSOD exists in most parts of cells, while MnSOD is only found in mitochondrial matrix; and both of them are able to effectively scavenge O_2_^.-^ and generate H_2_O_2_ (Li et al., 1995; Melov et al., 2001; Elchuri et al., 2005). H_2_O_2_ can be scavenged by GPX's (glutathione peroxidases) and peroxiredoxins (thioredox-independent peroxidases) (Chu et al., 2004; Kang et al., 2005). Studies have found that the levels of CuZnMOD and MnSOD are significantly lower in PAs compared to those of controls (Kurisaka et al., 2004; Yang et al., 2012; Ilhan et al., 2018). The abnormal activities of these antioxidant enzymes and non-enzymatic antioxidants are directly associated with carcinogenesis (Neumann et al., 2003; Chu et al., 2004; Harris et al., 2015). The transcription factor Nrf2 is pivotal to the antioxidant response, which is a sensor of oxidative stress in redox homeostasis, and is mainly located in the cytoplasm under basal conditions (Li and Kong, 2009; Furfaro et al., 2016a). When the upload of free radicals ROS/RNS is increased to cause oxidative stress, Nrf2 quickly translocates from cytoplasm into the nucleus to initiate the antioxidant response, protecting against oxidative/nitrative damages (Dhakshinamoorthy and Porter, 2004; Osburn et al., 2006; Mann et al., 2007; Pi et al., 2008). The Nrf2 signaling regulatory system contains at least four components, including Nrf2, Kelch-like ECH-associated protein 1 (Keap1), small musculoaponeurotic fibrosarcoma (Maf), and antioxidant response element (ARE) or electrophile responsive element (EpRE), which in combination are necessary for the antioxidant response (Kwak and Kensler, 2010; Furfaro et al., 2016; de la Vega et al., 2018). Nrf2 signaling pathways regulate multiple biological processes, including i) the expressions of antioxidant genes, ii) ubiquitin-proteasome system, iii) molecular chaperone/stress-response system, and iv) anti-inflammatory response (Kwak and Kensler, 2010; Furfaro et al., 2016). The accumulated evidence clearly demonstrates that Nrf2 signaling pathways are involved in 12 hallmarks of cancer, including sustained proliferative signaling, insensitivity to antigrowth signals, resistance to apoptosis, limitless replicative potential, sustained angiogenesis, tissue invasion and metastasis, metabolic reprogramming, avoiding immune destruction, tumor-promoting inflammation, genome instability, altered redox homeostasis, and proteotoxic stress (de la Vega et al., 2018). Thereby, any decreased capability of the antioxidant protective system in the redox homeostasis might cause more susceptibility to carcinogen toxicity, tumor inflammatory response, oxidative stress, and carcinogenesis (Yates and Kensler, 2007).

## Multiuomics Reveals Oxidative Stress-Related Pathway Alterations in PAs

Our multiomics studies in PAs (Zhan and Desiderio, 2010a; Long et al., 2019) clearly demonstrate oxidative stress-related pathway changes in PAs. For example, i) Nrf2-mediated oxidative stress response pathway is significantly changed in NFPAs with evidence of upregulation of key molecules [upregulated DEPs: GST (glutathione S-transferase) or GSTM2 (glutathione S-transferase mu 2), and ERP29 (endoplasmic reticulum protein 29], and downregulation of key molecules [downregulated DEPs: HSP22 (heat shock protein 22), HSP27, and HSP90 or GRP94 (94 kD glucose-regulated protein)] in this pathway. ii) Mitochondrial dysfunction pathway is significantly changed in NFPAs with evidence of upregulation of key molecules [upregulated DEPs: NDUFS8 (NADH ubiquinone oxidoreductase core subunit S8), COX6B (cytochrome c oxidase subunit 6B), CAT (catalase), β-secret2, and ATP5B (ATP synthase, H+ transporting mitochondrial F1 complex, beta subunit)], and downregulation of key molecules [downregulated DEPs: GPX4 (glutathione peroxidase 4), and ATP5A1] in this pathway. Mitochondrial dysfunction can increase ROS production in cancer cells to mediate tumor-related signaling pathways and activate pro-oncogenic signaling (Li and Zhan, 2019). iii) Oxidative phosphorylation pathway is significantly changed in NFPAs with evidence of upregulation of key molecules (upregulated DEPs: NDUFS8, COX6B, and ATP5B) in this pathway. Mitochondrial oxidative phosphorylation system contains mitochondrial complexes I, II, III, IV, and V, which are the major sites that produce endogenous ROS such as OH^.^ and O^−^_2_; among these, complexes I, II, and III play a crucial role in the generation of mitochondrial ROS, because the electrons tend to be leaky at complexes I and III, which results in an incomplete reduction of oxygen and thus generates a free radical such as superoxide radical (Li and Zhan, 2019). iv) Glutathione redox reaction I pathway is significantly changed in NFPAs with evidence of downregulation of key molecule (downregulated DEP: GPX4) in this pathway. GPX’s (glutathione peroxidases) are important components in the antioxidant defense system: the downregulation of GPX’s can decrease the capability of the antioxidant defense system. v) The superoxide radical degradation pathway is significantly changed in NFPAs with evidence of upregulation of key molecule (upregulated DEP: CAT) in this pathway. vi) Aryl hydrocarbon receptor signaling is significantly changed in NFPAs with evidence of upregulation of key molecules [upregulated DEP: GST; upregulated DEGs: HSPCA (heat shock protein 90 alpha family class A member 1), HSPCB (heat shock protein 90 alpha family class B member 1), ESR1 (estrogen receptor 1), and Bax (BCL2 associated X, apoptosis regulator)], and downregulation of key molecules [downregulated DEPs: HSP27, HSP90 or GRP94, and TGM2 (transglutaminase 2); downregulated DEG: ESR2 (estrogen receptor 2)] in this pathway. vii) Glucocorticoid receptor signaling is significantly changed in NFPAs with evidence of upregulation of key molecule [upregulated DEG: PI3K (phosphatidylinositol 3 kinase)], and downregulation of key molecules [downregulated DEGs: HSP70, c-Fos, CCL2 (C-C motif chemokine ligand 2), BCL2, PRL, and POMC (proopiomelanocortin)] in this pathway. viii) Corticotropin-releasing hormone signaling is significantly changed in NFPAs with evidence of upregulation of key molecules [upregulated DEGs: CALM (calmodulin), and IP3R)], and downregulation of key molecules [downregulated DEGs: ACTH, Nur77 (NR4A1 nuclear receptor subfamily 4 group A member 1), and c-FOS)] in this pathway. ix) Melatonin signaling is significantly changed in NFPAs with evidence of nitration of key molecule (PKA) in this pathway. x) Methylglyoxal degradation III pathway is significantly changed in NFPAs with evidence of upregulation of key molecules [upregulated DEPs: aldose reductase or AKR1B1(aldo-keto reductase family 1 member B)] in this pathway. xi) AMPK signaling is significantly changed in NFPAs with evidence of upregulation of key molecules [upregulated DEGs: PP2C (putative protein phosphatase), and PFK (phosphofructokinase)], and downregulation of key molecules [downregulated DEGs: PI3K, PKA, and PDK1 (pyruvate dehydrogenase kinase 1)] in this pathway. Thereby, these signaling pathway changes clearly demonstrate that the disturbance in redox homeostasis, the imbalance between generation and detoxification of free radicals ROS/RNS, results in oxidative stress and damage in human PAs. Recently, these findings are also confirmed with experiments in cell models and animal models, which demonstrate that increased mitochondrial fusion results in bigger mitochondria, increased ROS levels, and oxidative damage in PAs, and that Nrf2 signaling pathway is activated in PAs as an antioxidant response (Sabatino et al., 2018). Thus, it suggests that Nrf2 is the master regulator of the cellular antioxidant response (de la Vega et al., 2018).

## Nrf2-Mediated Oxidative Stress Response Signaling Pathways in PAs

**FIGURE 1:**
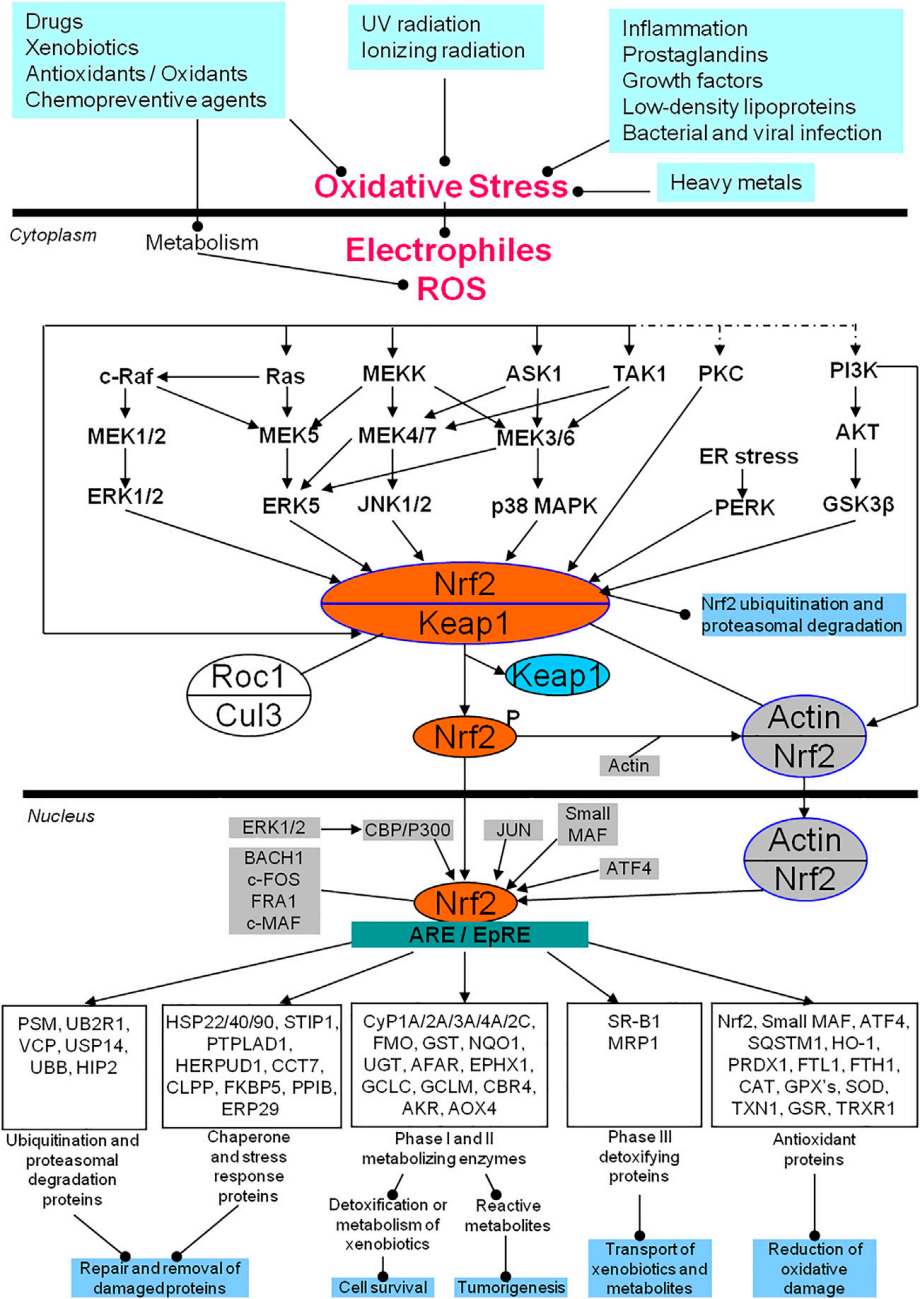
Nrf2-mediated oxidative stress response signaling pathways in human pituitary adenomas. AKR, Palmitoyltransferase; AKT, Protein kinase B; AOX4, Aldehyde oxidase 4; ARE, Antioxidant response element; ASK1, Apoptosis signal-regulating kinase 1; ATF4, Activating transcription factor 4; BACH1, Transcription regulator protein BACH1; CAT, catalase; CBP, CREB-binding protein; CBR4, carbonyl reductase 4; CCT7, T-complex protein 1 subunit eta; c-FOS, Proto-oncogene protein c-FOS; CLPP, Caseinolytic protease; Cul3, Cullin 3-based ubiquitin E3 ligase complex; Cyp, cytochrome P; EPHX1, Epoxide hydrolase 1; EpRE, Electrophile responsive element; ER, endoplasmic reticulum; ERK: Extracellular signal-related kinase; ERP29: endoplasmic reticulum protein 29; FKBP5, FK506-binding protein 5; FMO, Dimethylaniline monooxygenase [N-oxide-forming]; FRA1, Fos-related antigen 1; FTH1, Ferritin heavy polypeptide 1; FTL1, ferritin light polypeptide; GCLC, glutamate-cysteine ligase catalytic subunit; GCLM, glutamate-cysteine ligase modifier subunit; GPX's, Glutathione peroxidases; GSK3β, glycogen synthase kinase 3β; GSR, glutathione reductase; GST, glutathione S-transferase; HERPUD1, Homocysteine-responsive endoplasmic reticulum-resident ubiquitin-like domain member 1 protein; HIP2, Ubiquitin-conjugating enzyme E2 K; HO-1, heme oxygenase 1; HSP22/40/90, heat shock proteins 22, 40 and 90; JNK, Jun N-terminal kinase; Keap1, Kelch-like ECH-associated protein 1; Maf, Musculoaponeurotic fibrosarcoma; MAPK, Mitogen-activated protein kinase; MEK, Mitogen-activated protein kinase kinase (MAPKK); MEKK, Mitogen-activated protein kinase kinase kinase (MAPKKK); MRP1, multidrug-resistant protein-1; NQO1, NAD(P)H:quinine oxidoreductase 1; Nrf2, Nuclear factor erythroid 2 p45-related factor 2; PERK: the double-stranded RNA (PKR)-activated protein kinase-like eukaryotic initiation factor 2 kinase; PI3K, Phosphatidylinositol 3 kinase; PKC, protein kinase C; PPIB, Peptidyl-prolyl cis-trans isomerase B; PRDX1, peroxiredoxin 1; PSM: multiple subunits of the 20S proteasome; PTPLAD1, 3-hydroxyacyl-CoA dehydratase 3; c-Raf, RAF proto-oncogene serine/threonine-protein kinase; Ras, GTPase Ras; ROS, reactive oxygen species; SOD, Superoxide dismutase; SQSTM1, sequestosome-1 protein; SR-B1, Scavenger receptor class B member 1; STIP1, stress induced phosphoprotein 1; TAK1, TGF beta-Activated Kinase 1; TXN1: thioredoxin; TRXR1, thioredoxin reductase 1; UBB, Polyubiquitin-B; UB2R1, Ubiquitin-conjugating enzyme E2 R1; UGT, UDP glucuronosyl transferase; USP14, ubiquitin-specific peptidase 14; and VCP, valosin-containing protein. Modified from Zhan X et al. (2010) (Zhan and Desiderio, 2010a), copyright permission from BioMed Central publisher open-access article, copyright 2010; and modified from Long Y et al. (2019) (Long et al., 2019), copyright permission from Frontiersin publisher open-access article, copyright 2019.

Nrf2 signaling pathway in response to oxidative stress is shown (Figure 1). Multiple *in vivo* and *in vitro* environmental factors, including inflammatory cytokines, prostaglandins, growth factors, low-density lipoproteins, bacterial and viral infection, heavy metals, ultraviolet (UV) radiation, ionizing radiation, drugs, xenobiotics, antioxidants, oxidants, and chemopreventive agents, cause the increased upload of free radicals ROS/RNS and electrophiles to result in oxidative stress (Hetland et al., 2020; Mehnati et al., 2020). The increased ROS or electrophiles will activate the Nrf2/Keap1 complex in the cytoplasm through ERK1/2, ERK5, JNK1/2, p38 MAPK, PKC, and PI3K-AKT signaling pathways, and these signaling pathways will communicate with each other (Roy Chowdhury et al., 2014; Tian et al., 2014; Wang K.-C. et al., 2019). The activated Nrf2 is phosphorylated and separated from Keap1 (Hambright et al., 2015; Sánchez-Martín et al., 2020). The separated and phosphorylated Nrf2 quickly translocates into the nucleus to interact with ARE or EpRE, which will initiate at least five types of gene expressions to exert the corresponding biological functions (Furfaro et al., 2016; Sánchez-Martín et al., 2020): i) reduction of the oxidative damage via antioxidant proteins such as NRF2, small MAF, ATF4, SQSTM1, HO-1, PRDX1, FTL, FTH1, CAT, GPX’s, SOD, TXN, GSR, and TRXR1 (Sun et al., 2019; Saad El-Din et al., 2020; Yu et al., 2020); ii) detoxification and metabolism of xenobiotics to regulate cell survival, or production of reactive metabolites to promote tumorigenesis via phase I and II metabolizing enzymes such as CYP1A/2A/3A/4A/2C, FMO, GST, NQD, UGT, AFAR, EPHX1, GCLC, GCLN, CBR4, AKR, and AOX4 (Zhao et al., 2015; Huang et al., 2018); iii) transportation of xenobiotics and metabolites via phase III detoxifying proteins such as SR-B1 and MRP1 (Sivils et al., 2013; Lubelska et al., 2016); iv) repairment and removal of the damaged proteins via chaperone and stress response proteins such as HSP22/40/90, STIP1, PTPLAD1, HERPUD1, CCT7, CLPP, FKBP5, PPIB, and ERP29 (Niture and Jaiswal, 2010; Sahin et al., 2012); and v) repairment and removal of the damaged proteins via ubiquitination and proteasomal degradation proteins such as PSM, UB2R1, VCP, USP14, UBB, and HIP2 (Liu et al., 2019; Song et al., 2019). This clearly demonstrates that while the Nrf2-mediated oxidative stress response signaling pathways are regulated by multiple factors, Nrf2 is the essential component. In the cytoplasm, Keap1, the main regulator of Nrf2, is a substrate adaptor protein for the Cul3-Keap1-E3 ligase complex that ubiquinates Nrf2, marking it for proteasomal degradation in the cytoplasm under basal conditions (Baird and Yamamoto, 2020; Dayalan Naidu and Dinkova-Kostova, 2020). To reduce its inhibitory effects on Nrf2, Keap1 can be ubiquitinated for degradation, leading to an increase in Nrf2 phosphorylation (activation) (Villeneuve et al., 2010). The phosphorylated Nrf2 can then interact with actin to form an Nrf2/actin complex that then translocates into the nucleus. After Nrf2 translocates into the nucleus, there are additional regulatory systems in place that include multiple factors such as ATF4, JUN, ERK1/2-CBP/P300, small MAF, BACH1, c-FOS, FRA1, and c-MAF, to influence the binding of Nrf2 and ARE/EpRE. The detailed regulatory mechanism system of Nrf2 has been extensively reviewed (Kwak and Kensler, 2010; Hybertson et al., 2011; Furfaro et al., 2016a; Lu et al., 2016; Menegon et al., 2016; Taguchi and Yamamoto, 2017; Bellezza et al., 2018; Chen and Maltagliati, 2018; de la Vega et al., 2018; Ryoo and Kwak, 2018; Sajadimajd and Khazaei, 2018; Cloer et al., 2019; Cuadrado et al., 2019; Qin et al., 2019) response signaling pathways have also been studied in pituitaries and PAs. One study shows that Nrf2, phosphorylated Nrf2 (p-Nrf2) protein, and mRNA expressions are increased in PAs, and the Nrf2 downstream effector HO-1 is also increased in PAs (Sabatino et al., 2018). This clearly demonstrates the activation of the Nrf2 signaling pathway, likely causing the extensive surviving capability of pituitary tumor cells. The Nrf2/PTEN-induced putative kinase protein 1 (PINK1)/Parkin pathway and mitophagy are activated in T-2 toxin-induced toxicities in rat pituitary GH3 cells (Deyu et al., 2018). Antioxidants N-acetylcysteine (NAC) and vitamin E can decrease the expressions of Nrf2 and HO-1 in rat pituitaries (Prevatto et al., 2017). Genetically induced Nrf2 overexpression in melanoma cells promotes tumor growth and increases antioxidant defense in malignant cells, which can be inhibited by anticancer agent pterostilbene (Pter, a natural dimethoxylated analog of resveratrol) through the downregulation of pituitary production of ACTH, plasma corticosterone, and the glucocorticoid receptor- and Nrf2-dependent antioxidant defense systems in growing melanomas (Benlloch et al., 2016). Irradiation can result in oxidative damage in C57/BL6 mice via activation of Nrf2 and HO-1 expressions, which can be blocked by antioxidant agent pituitary adenylate cyclase-activating polypeptide 38 (PACAP38) through inhibiting Nrf2 expression (Li et al., 2019). Chronic restraint stress (CRS) and acute restraint stress (ARS) can upregulate the mRNA expressions of oxidative stress molecules (gp91phox, iNOS, and Nrf2) and inflammation-related molecules (IL-1β, IL-6, TNFα, and TLR4) in the mouse hypothalamus, which can be alleviated by Iptakalim (Ipt), an ATP-sensitive potassium (K-ATP) channel opener (Zhao et al., 2017). The loss-of-function mutations of the aryl hydrocarbon receptor-interacting protein gene (AIP) are well-recognized in PAs (Hernández-Ramírez et al., 2018). The aryl hydrocarbon receptor signaling is also revealed by multiomics as an oxidative stress-related signaling pathway in PAs (Long et al., 2019). Further studies show that AIP interacts with antioxidants, chaperone and stress response-related proteins, and cytoskeletal proteins, including HSPA5, HSPA9, HSP90AA1, HSP90AB1, HSPA8, SOD1, TUBB, TUBB2A, and NME1; AIP variants show the impaired interaction of AIP with HSPA8, HSP90AB1, NME1, SOD1, TUBB, and TUBB2A; AIP-mutated PAs show the reduced expression of TUBB2A (Cuadrado et al., 2019). The levels of MnSOD and total antioxidant capability (TAC) are significantly decreased in GH-secreting PAs (Ilhan et al., 2018). The frequencies of micronuclei (MN), nucleoplasmic bridges, nuclear buds, apoptotic and necrotic cells, and plasma 8-hydroxy-2′-deoxyguanosine (8-OHdG) levels in peripheral blood lymphocytes are significantly increased in PRL-secreting PAs, which indicates the increased oxidative damage in PRL-secreting PAs (Bitgen et al., 2016). Oxidative stress and mitochondrial dysfunction have been revealed by multiple proteomics and nitroproteomics studies in human PAs (Zhan and Desiderio, 2010a; Zhan et al., 2013; Zhan et al., 2014a; Zhan et al., 2014b; Wang X. et al., 2015; Long et al., 2019). Also, tumor inflammation is an important pathophysiological characteristic in human PAs, which is always tightly associated with oxidative stress and chronic inflammation. The relationship among age-related disease, chronic inflammation, and oxidative stress has also been discussed (Pizza et al., 2011; Liguori et al., 2018; Qian et al., 2019). Oxidative stress is also involved in the processes of anti-proliferative effect and cell death induced by dopamine in the pituitary tumor cells via dopamine D2 receptors through p38 MAPK, and ERK pathways (An et al., 2003). Therefore, oxidative stress and antioxidative stress response extensively exist in PA pathogenesis. Nrf2, as the core of oxidative stress response, could be the novel target used to develop effective therapeutic agents for human PAs (Kwak and Kensler, 2010; Furfaro et al., 2016; de la Vega et al., 2018).

## Therapeutic Status Targeting Nrf2 Signaling Pathways in Cancers

Nrf2 signaling, as the heart of oxidative stress response, is extensively related to cancer pathogenesis, which has attracted tremendous attention as possible anticancer therapeutic target. Nrf2 signaling-based anticancer therapeutic studies have been extensively carried out in multiple cancers, including acute myeloid leukemia, gallbladder cancer, renal carcinoma, pancreatic cancer, melanoma, hepatocellular carcinoma, lung cancer, colon cancer, ovarian cancer, breast cancer, esophageal cancer, and glioblastoma (Table 1). i) In acute myeloid leukemia, studies found that Nrf2 activators [dimethyl fumarate (DMF), tert-butylhydroquinone, or carnosic acid] and vitamin D derivatives can cooperatively induce acute myeloid leukemia cell differentiation to inhibit leukemia progression in a xenograft mouse model via activating the Nrf2/ARE signaling pathway (Nachliely et al., 2019). Novel pyrazolyl hydroxamic acid derivative (4f) can inhibit Nrf2 activity to induce apoptosis of human acute myeloid leukemia cells (Zhang et al., 2017). ii) In gallbladder cancer, one study found that atypical protein kinase Cι (aPKCι) can promote gallbladder tumorigenesis and chemoresistance of anticancer agent gemcitabine by competing with Nrf2 for binding to Keap1, implying that inhibiting the aPKC1-Keap1-Nrf2 axis might overcome drug resistance for the treatment of gallbladder cancer (Tian et al., 2019). iii) In renal carcinoma, one study found that the natural product chitosan oligosaccharide (COS) can inhibit human renal carcinoma cell proliferation *in vitro* and *in vivo* by promoting the expressions of Nrf2 and Nrf2 target genes such as HO-1, the modifier subunit of glutamate cysteine ligase, solute carrier family 7 member 11, glucose-regulated protein 78, protein RNA-like endoplasmic reticulum kinase, and cytochrome C. (Zhai et al., 2019). iv) In pancreatic cancer, one study found that anticancer agent resveratrol enhances the sensitivity of pancreatic cancer cells to gemcitabine via suppressing NAF-1 (nutrient-deprivation autophagy factor-1) expression, inducing ROS accumulation, and activating Nrf2 signaling pathways (Cheng et al., 2018). v) In melanoma, the co-treatment of Nrf2 inhibitor (brusatol, BR) and UVA irradiation can effectively inhibit melanoma growth by regulating AKT-Nrf2 pathway (Wang et al., 2018). vi) In hepatocellular carcinoma, one study found that potential Nrf2 inhibitors can sensitize chemotherapy drugs in hepatocellular carcinoma (Tian et al., 2018). Cordycepin (CA) can activate the Nrf2/HO-1/NF-κB pathway for its anti-hepatocarcinoma effect in N-nitrosodiethylamine (NDEA)-induced mouse hepatocellular carcinomas (Zeng et al., 2017). The novel indazolo[3,2-b]quinazolinone (IQ) derivatives, IQ-7 and IQ-12, can induce apoptosis of human hepatoma cells Hep3B and inhibit the Nrf2/ARE signaling pathway in Hep3B cells, and IQ-7 is suggested as a degree of specificity against cancer cells (Zhang et al., 2016). Also, dibenzoylmethane (DBM) can protect against carbon tetrachloride (CCl4)-induced liver injury by activating Nrf2 signaling via JNK, AMPK, and calcium signaling (Cao et al., 2017). vii) In lung cancer, one study found that the potent anticancer agent isodeoxyelephantopin can induce protective autophagy in lung cancer cells via the Nrf2-p62-keap1 pathway (Wang et al., 2017). The Nrf2 activators, DMF and the synthetic oleanane triterpenoids, activate the Nrf2 pathway as well as regulate different subsets of Nrf2 target genes and Nrf2-independent genes in lung cancer (Chian et al., 2014; To et al., 2015). viii) In colon cancer, one study found that anticancer agent sulforaphane (SFN) can activate Nrf2 signaling to suppress human colon cancer (Johnson et al., 2017). Also, taxifolin (TAX) can induce antioxidant response pathway and enhance level of Nrf2 protein, and act as effective chemopreventive agent capable of modulating inflammation in colon cancer (Manigandan et al., 2015). ix) In ovarian cancer, one study found that Nrf2 can mediate the response of cancer cells to the anti-HER2 drugs, trastuzumab and pertuzumab, in ovarian cancer cells (Khalil et al., 2016). Also, activation of Nrf2 pathway in ovarian cancer seems to be related to Keap1 mutations within highly conserved domains of Keap1 gene and that Nrf2 may serve as an important therapeutic target for novel drugs capable of preventing or reversing resistance to chemotherapy in ovarian cancer (Konstantinopoulos et al., 2011). x) In breast cancer, Nrf2 serves as a key regulator in chemotherapeutic resistance under hypoxia through ROS-Nrf2-GCLC-GSH pathway and can be a potential treatment for hypoxia induced drug resistance in breast cancer cells (Song et al., 2011; Syu et al., 2016). xi) In esophageal cancer, C-28 methyl ester of 2-cyano-3,12-dioxoolean-1,9-dien-28-oic acid (CDDO-Me) can protect the cells against oxidative stress via inhibition of ROS generation, while CDDO-Me at low micromolar concentrations induces apoptosis by increasing ROS and decreasing intracellular glutathione levels in esophageal squamous cancer cells (Wang Y. Y. et al., 2015). xii) In glioblastoma, there are many potent anti-cancer agents targeting Nrf2 signaling for chemotherapy and chemoresistance in glioblastoma (Zhu et al., 2014). xiii) In osteosarcoma, the bioengineered Nrf2-siRNA can effectively interfere with the Nrf2 signaling pathway to improve chemosensitivity of human cancer cells (Li et al., 2018). Moreover, the PIM (proviral integration site for moloney murine leukemia virus) kinase inhibitors can reduce Nrf2 signaling and increase ROS to kill hypoxic tumor cells such as prostate cancer cells (PC4-LN4), colon cancer cells (HCT-116), and breast cancer cells (MB-MDA-231 and MB-MDA-231-ARE-Luc) (Warfel et al., 2016). One study shows that proteasome biogenesis is dependent on the Nrf2 transcriptional factor, thus proteasome inhibitors have been actively developed as potential anticancer drugs (Albornoz et al., 2019). Gallic acid (GA), Z-ligustilide (LIG), and senkyunolide A (SA) can individually or cooperatively target Nrf2/ARE pathway to prevent cancer (Liu et al., 2018). Therefore, it can be said that Keap1-Nrf2 signaling pathways have different roles at different stages of cancer (Leinonen et al., 2014; Furfaro et al., 2016; de la Vega et al., 2018). Multiple Nrf2 or Keap1 inhibitors have been reported; and some of them are in the stages of pre- and clinical trial towards the Nrf2 signaling for cancers. For example, sulforaphane can target Nrf2 and the Nrf2 target genes NQO1 and GCLC to prevent oral cancer, and a preclinical trail has been performed to study its chemopreventive activity for oral cancer (Bauman et al., 2016). A single centre, single arm prospective phase II clinical trial has been performed for phytosome complex of curcumin targeting Nrf2 signaling as a the complementary therapy of gemcitabine on pancreatic cancer (Pastorelli et al., 2018). However, none of these Nrf2 or Keap1 inhibitors have currently entered into real clinical applications, which suggests that the sole inhibition of Nrf2 might not be sufficient for anticancer. A rational combination of Nrf2 inhibitors with other chemical agents would be a better strategy to treat cancers (Zhang et al., 2019).

**TABLE 1:** Current research status of therapeutic potentials targeting Nrf2-mediated oxidative stress response signaling pathways in different cancers.

Cancer type	Experimental model	Chemical reagents or potential drugs	Possible mechanisms	References
Acute myeloid leukemia	Acute myeloid leukemia cells in a xenograft mouse model	Nrf2 activators: dimethyl fumarate (DMF), tert-butylhydroquinone, or carnosic acid	Cooperate with vitamin D derivatives to induce acute myeloid leukemia cell differentiation to inhibit leukemia progression in a xenograft mouse model via activating the Nrf2/ARE signaling pathway	Nachliely et al. (2019)
	Human acute myeloid leukemia cells	Novel pyrazolyl hydroxamic acid derivative (4f)	Inhibit Nrf2 activity to induce apoptosis of human acute myeloid leukemia cells	Zhang et al. (2019)
Gallbladder cancer	Gallbladder cancer cells	The aPKCι inhitors, Nrf2 activators, or gemcitabine	Atypical protein kinase Cι (aPKCι) can promote gallbladder tumorigenesis and chemoresistance of anticancer agent gemcitabine by competing with Nrf2 for binding to Keap1, implying that inhibiting the aPKCι-Keap1-Nrf2 axis might overcome drug resistance for the gallbladder cancer treatment	Tian et al. (2019)
Renal carcinoma	Human renal carcinoma cells	Chitosan oligosaccharide (COS)	Inhibit human renal carcinoma cell proliferation *in vitro* and *in vivo* by promoting the expressions of Nrf2 and Nrf2 target genes such as HO-1, the modifier subunit of glutamate cysteine ligase, solute carrier family 7 member 11, glucose-regulated protein 78, protein RNA-like endoplasmic reticulum kinase, and cytochrome C,etc.	Zhai et al. (2019)
Pancreatic cancer	Pancreatic cancer cells	Resveratrol	Enhance the sensitivity of pancreatic cancer cells to gemcitabine via suppressing NAF-1 expression, inducing ROS accumulation, and activating Nrf2 signaling pathways	Cheng et al. (2018)
Melanoma	Melanoma cells	Nrf2 inhibitor: Brusatol (BR)	The co-treatment of brusatol and UVA irradiation can effectively inhibit melanoma growth by regulating the AKT-Nrf2 pathway	Wang et al. (2018)
Hepatocellular carcinoma	Hepatocellular carcinoma (HCC) cells	Vitamin C (VC), all-trans retinoic acid (ATRA), ochratoxin A (OTA), bexarotene, flavonoids (including brusatol, luteolin, apigenin and chrysin), ruthenium (Ru) metal complexes, ursolic acid (UA), halofuginone, trigonelline, quercetin, and isoniazid	Sensitize chemotherapy drugs in hepatocellular carcinoma	Tian et al. (2018)
	Mouse hepatocellular carcinoma model	Cordycepin (CA)	Activate the Nrf2/HO-1/NF-κB pathway for its anti-hepatocarcinoma effect in N-nitrosodiethylamine (NDEA)-induced mouse hepatocellular carcinomas	Zeng et al. (2017)
	Hep3B (human hepatoma cell) and HL-7702 (normal human liver cell) cell lines	Novel indazolo[3,2-b] quinazolinone (IQ) derivatives: IQ-7 and IQ-12	Induce apoptosis and inhibit the Nrf2/ARE signaling pathway in Hep3B cells, and IQ-7 was suggested a degree of specificity against cancer cells.	Zhang et al. (2016)
	Liver injury mouse model	Dibenzoylmethane (DBM)	Protect against carbon tetrachloride (CCl4)-induced liver injury by activating Nrf2 signaling via JNK, AMPK, and calcium signaling	Cao et al. (2017)
Lung cancer	Lung cancer cells	The potent anticancer agent: Isodeoxyelephantopin	Induce protective autophagy in lung cancer cells via the Nrf2-p62-keap1 pathway	Wang et al. (2017)
	RAW 264.7 mouse macrophage-like cells, in VC1 lung cancer cells, and in the A/J model of lung cancer	Two clinically relevant classes of Nrf2 activators: DMF, and the synthetic oleanane triterpenoids –C-28 methyl ester of 2-cyano-3,12-dioxoolean-1,9-dien-28-oic acid (CDDO)-Imidazolide (CDDO-Im) and CDDO-Methyl ester (CDDO-Me)	Activate the Nrf2 pathway as well as regulate different subsets of Nrf2 target genes and Nrf2-independent genes	Chian et al. (2014) and To et al. (2015)
Colon cancer	SFN-treated human colon cancer cells and non-transformed colonic epithelial cells	Anticancer agent: Sulforaphane (SFN)	Regulate the activity of antioxidant and the detoxification of carcinogens via Nrf2 signaling to suppress human colon cancer	Johnson et al. (2017)
	1, 2-dimethyl hydrazine (DMH)-induced mouse colon model	Taxifolin (TAX)	Induce antioxidant response pathway, enhance level of Nrf2 proteins, and act as effective chemopreventive agent capable of modulating inflammatory	Manigandan et al. (2015)
Ovarian cancer	Human ovarian cancer cell lines: PEO4, OVCAR4, and SKOV3	Anti-HER2 drugs: Trastuzumab and Pertuzumab	HER2 targeting by antibodies inhibited growth in association with persistent ROS generation, glutathione (GSH) depletion, reduction in NRF2 levels, and inhibition of NRF2 function in ovarian cancer cell lines	Khalil et al. (2016)
	Human epithelial ovarian cancer (EOC) cell lines	Keap1 mutation reagent	Activation of Nrf2 pathway in EOC seems to be related to Keap1 mutations within highly conserved domains of the Keap1 gene; and Nrf2 may serve as an important therapeutic target for novel drugs capable of preventing or reversing resistance to chemotherapy in EOC	Konstantinopoulos et al. (2011)
Breast cancer	Breast cancer cells, and mouse model	Target antioxidant enzymes: GCLC and GCLM	Nrf2 serves as a key regulator in chemotherapeutic resistance under hypoxia through ROS-Nrf2-GCLC-GSH pathway, and can be a potential treatment for hypoxia-induced drug resistance in breast cancer cells.	Syu et al. (2016) and Song et al. (2011)
Esophageal cancer	Esophageal squamous cancer cells (ESCC): Ec109 and KYSE70 cells	CDDO-Me	Protects the cells against oxidative stress via inhibition of ROS generation, while CDDO-Me at low micromolar concentrations induces apoptosis by increasing ROS and decreasing intracellular glutathione levels	Wang X. et al. (2015)
Glioblastoma	Glioblastoma cells	Potential anti-cancer agents	Targeting Nrf2 signaling for chemotherapy and chemoresistance	Zhu et al. (2014)
Osteosarcoma	Human osteosarcoma 143B and MG63 cells	The bioengineered Nrf2-siRNA	Interfere with the Nrf2 signaling pathway to reduce the expression of NRF2-regulated oxidative enzymes and lead to higher intracellular ROS levels; knocking down NRF2 with bioengineered siRNA agent improves chemosensitivity of cancer cells, which is related to the suppression of NRF2-regulated efflux ABC transporters.	Li et al. (2018)
Other cancers	prostate cancer cell PC4-LN4; colon cancer cell HCT-116; breast cancer cells MB-MDA-231 and MB-MDA-231-ARE-Luc	PIM kinases inhibitors	Inhibit Nrf2 signaling and increase ROS to kill hypoxic tumor cells in a HIF-1-independent manner by controlling its cellular localization	Warfel et al. (2016)
	Mammalian cancer cells	Proteasome inhibitors	In response to proteasome inhibition, several responses are activated, such as the ALP, proteaphagy, the transcriptional upregulation of the autophagy Ubreceptor p62/SQSTM1, and proteasome genes, by Nrf1 and Nrf1/Nrf2 transcription factors, respectively.	Albornoz et al. (2019)
	Mouse epidermal cells (JB6 P+),	Gallic acid (GA), Z-ligustilide (LIG), and senkyunolide A (SA)	GA, LIG, and SA in Si-Wu-Tang (SWT) can individually or cooperatively target the Nrf2/ARE pathway to prevent cancer.	Liu et al. (2018)

ALP, Autophagic-Lysosomal Pathway; ATRA, All-trans retinoic acid; BR, Brusatol; CA, Cordycepin; CDDO, C-28 methyl ester of 2-cyano-3,12-dioxoolean-1,9-dien-28-oic acid; COS, Chitosan oligosaccharide; DBM, Dibenzoylmethane; DMF, dimethyl fumarate; GA, Gallic acid; IQ, Indazolo[3,2-b] quinazolinone; LIG, Z-ligustilide; OTA, Ochratoxin A; PIM, The Proviral Integration site for Moloney murine leukemia virus; Ru, Ruthenium; SA, Senkyunolide A; SFN, Sulforaphane; TAX, Taxifolin; UA, Ursolic acid; VC, vitamin C.

## Potential of Targeting Nrf2 Signaling as New Therapeutic Strategy for PAs

As described above, many omics studies in human PA tissues and experimental studies in PA cells and animal models demonstrate that oxidative stress and oxidative damage is the important hallmark of PA pathogenesis. Nrf2-mediated oxidative stress response signaling pathways are at the heart of oxidative stress response, and many chemical agents targeting Nrf2 signaling pathways have been developed and tested as potential anticancer drugs for different cancers. This clearly demonstrates the potential of targeting Nrf2 signaling pathways as new therapeutic strategies for PAs. However, the use of Nrf2 signaling as a therapeutic target for PAs has not been studied. We strongly believe that the Nrf2-mediated oxidative stress response signaling pathways are the promising targets for novel therapeutic strategies for PAs. Furthermore, MAPK signaling pathways including ERK, JNK, and p38 MAPK clearly regulate Nrf2 signaling (Figure 1). Moreover, MAPK signaling pathways have been recognized as potential therapeutic targets for PAs (Lu et al., 2019). The combined use of Nrf2 inhibitors targeting Nrf2 signaling and ERK inhibitors [e.g., somatostatin analogs pasireotide (SOM230) and octreotide (OCT), or dopamine] plus *p*38 activators (e.g., cabergoline, bromocriptine, and fulvestrant) or JNK activators (e.g., ursolic acid, UA) targeting MAPK signaling pathways (Lu et al., 2019) might produce better anti-tumor effects on PAs. In addition, oxidative stress is tightly associated with mitochondrial dysfunctions, both operate in PAs (Zhan and Desiderio, 2010a; Li and Zhan, 2019; Long et al., 2019). Some drugs targeting mitochondria are also recognized as a therapeutic strategy for PAs, including pyrimethamine, temozolomide, melatonin, melatonin inhibitors, gossypol acetate, 18 beta-glycyrrhetinic acid, T-2 toxin, Yougui pill, cyclosporine A, grifolic acid, paeoniflorin, and dopamine agonists (Li and Zhan, 2019). Therefore, the combined use of Nrf2 inhibitors targeting Nrf2 signaling and drugs targeting metochondria could be another way to generate better anti-tumor effects on PAs.

## Conclusion

Pituitary adenoma (PA) is a common and important disease that occurs in the hypothalamic-pituitary-target organ axis system and seriously affects human endocrine system and health. The imbalance between oxidative stress and the antioxidant defense system is an important pathophysiological characteristic in PAs, which has been evidenced by many omics analysis in PA tissues and experimental studies in PA cells and animal models. Nrf2 signaling is at the heart of oxidative stress response signaling pathways. Multiple anticancer agents targeting Nrf2-mediated oxidative stress response pathways have been developed and tested as potential therapeutic drugs for different cancers. However, Nrf2 signaling and targeting Nrf2 signaling as a therapeutic strategy has not yet been extensively studied in PAs. We strongly recommend the emphasis on in-depth studies of Nrf2 signaling and potential therapeutic agents targeting Nrf2 signaling pathways in PAs. Furthermore, the combined use of Nrf2 inhibitors targeting Nrf2 signaling and ERK inhibitors plus *p*38 activators or JNK activators targeting MAPK signaling pathways, or drugs targeting mitochondria dysfunction pathway might produce better anti-tumor effects on PAs.

## Glossary

ACTH: Adrenocorticotropin
AFAR: aldo-keto reductase family 7 member A2
AIP: Aryl hydrocarbon receptor interacting protein gene
AKR: Palmitoyltransferase AKT
AKR1B1: aldo-keto reductase family 1 member B
AKT: Protein kinase B
AOX4: Aldehyde oxidase 4
AP-1: Activator protein-1
aPKCι: Atypical protein kinase Cι
ARE: Antioxidant response element
ARS: Acute restraint stress
ASK1: Apoptosis signal-regulating kinase 1
ATF4: Activating transcription factor 4
ATP5A1: ATP synthase subunit alpha, mitochondrial
ATP5B: ATP synthase, H+ transporting mitochondrial F1 complex, beta subunit
BACH1: Transcription regulator protein BACH1
Bax: BCL2 associated X, apoptosis regulator
BCL2: BCL2 apoptosis regulator
BR: Brusatol
CALM: calmodulin
CAT: catalase
CBP: CREB-binding protein
CBR4: carbonyl reductase 4
CCL2: C-C motif chemokine ligand 2
CCT7: T-complex protein 1 subunit eta
c-FOS: Proto-oncogene protein c-FOS
CLPP: Caseinolytic protease
COS: Chitosan oligosaccharide
COX6B: cytochrome c oxidase subunit 6B
c-MAF: (MAF) MAF bZIP transcription factor
c-Raf: RAF proto-oncogene serine/threonine-protein kinase
CRS: Chronic restraint stress
Cul3: Cullin 3-based ubiquitin E3 ligase complex
Cyp: cytochrome P
CYP1A: cytochrome P450 family 1 subfamily A
CYP2A: cytochrome P450 family 2 subfamily A
CYP2C: cytochrome P450 family 2 subfamily C
CYP3A: cytochrome P450 family 3 subfamily A
CYP4A: cytochrome P450 family 4 subfamily A
DBM: Dibenzoylmethane
DEG: Differentially expressed gene
DEP: Differentially expressed protein
DMF: Dimethyl fumarate
EPHX1: Epoxide hydrolase 1
EpRE: Electrophile responsive element
ER: endoplasmic reticulum
ERK: Extracellular signal-related kinase
ERK1/2: mitogen-activated protein kinase
ERK5: mitogen-activated protein kinase
ERP29: endoplasmic reticulum protein 29
ESR1: estrogen receptor 1
ESR2: estrogen receptor 2
FKBP5: FK506-binding protein 5
FMO: Dimethylaniline monooxygenase [N-oxide-forming]
FPA: Functional pituitary adenoma
FRA1: Fos-related antigen 1
FSH: Follicle-stimulating hormone
FSHRH: Follicle-stimulating hormone-releasing hormone
FTH1: Ferritin heavy polypeptide 1
FTL1: ferritin light polypeptide
GA: Gallic acid
GCLC: glutamate-cysteine ligase catalytic subunit
GCLM: glutamate-cysteine ligase modifier subunit
GH: Growth hormone
GPX4: glutathione peroxidase 4
GPX's: Glutathione peroxidases
GRP94: 94 kD glucose-regulated protein
GSK3β: glycogen synthase kinase 3β
GSR: glutathione reductase
GST: glutathione S-transferase
GSTM2: glutathione S-transferase mu 2
HERPUD1: Homocysteine-responsive endoplasmic reticulum-resident ubiquitin-like domain member 1 protein
HIP2: Ubiquitin-conjugating enzyme E2 K
HO-1: heme oxygenase 1
HSP22/40/90: heat shock proteins 22, 40 and 90
HSP27: heat shock protein 27
HSP70: (HSPA4) heat shock protein family A member 4
HSP90: heat shock protein 90
HSP90AA1: heat shock protein 90 alpha family class A member 1
HSP90AB1: heat shock protein 90 alpha family class B member 1
HSP94: heat shock protein 94
HSPA5: heat shock protein family A (Hsp70) member 5
HSPA8: heat shock protein family A (Hsp70) member 8
HSPA9: heat shock protein family A (Hsp70) member 9
HSPCA: (HSP90AA1) heat shock protein 90 alpha family class A member 1
HSPCB: (HSP90AB1) heat shock protein 90 alpha family class B member 1
IL-1β: interleukin 1 beta
IL-6: interleukin 6
iNOS: Inducible nitric synthase
IP3R: (ITPR1) inositol 1,4,5-trisphosphate receptor type 1
Ipt: Iptakalim
JNK: Jun N-terminal kinase
JNK1: (MAPK8) mitogen-activated protein kinase 8
JNK 2: (MAPK9) mitogen-activated protein kinase 9
JUN: Jun proto-oncogene, AP-1 transcription factor subunit
K-ATP: ATP-sensitive potassium
Keap1: Kelch-like ECH-associated protein 1
LH: Luteinizing hormone
LHRH: Luteinizing hormone-releasing hormone
LIG: Z-ligustilide
Maf: Musculoaponeurotic fibrosarcoma
MAPKs: Mitogen-activated protein kinases
MEK: Mitogen-activated protein kinase kinase (MAPKK)
MEKK: Mitogen-activated protein kinase kinase kinase (MAPKKK)
MN: Micronucleus
MRP1: multidrug-resistant protein-1
NAC: N-acetylcysteine
NAF-1: Nutrient-deprivation autophagy factor-1
NDEA: N-nitrosodiethylamine
NDUFS8: (NADH) ubiquinone oxidoreductase core subunit S8
NF-kB: Nuclear factor kB
NFPA: Nonfunctional pituitary adenoma
NME1: NME/NM23 nucleoside diphosphate kinase 1
NO: Nitric oxide
NQO1: NAD(P)H:quinine oxidoreductase 1
Nrf2: Nuclear factor erythroid 2 p45-related factor 2
Nur77: (NR4A1) nuclear receptor subfamily 4 group A member 1
O2.-: Superoxide radical
8-OHdG: 8-hydroxy-2’-deoxyguanosine
OCT: octreotide
OH: hydroxyl radical
ONOO-: Peroxynitrite anion
PA: Pituitary adenoma
PACAP38: Pituitary adenylate cyclase-activating polypeptide 38
PDK1: pyruvate dehydrogenase kinase 1
PERK: the double-stranded RNA (PKR)-activated protein kinase-like eukaryotic initiation factor 2 kinase
PFK: phosphofructokinase
PI3K: Phosphatidylinositol 3 kinase
PINK1: PTEN-induced putative kinase protein 1
PKA: cAMP dependent protein kinase
PKC: protein kinase C
POMC: proopiomelanocortin
PPIB: Peptidyl-prolyl cis-trans isomerase B
PP2C: putative protein phosphatase
PRDX1: peroxiredoxin 1
PRL: Prolactin
PSM: multiple subunits of the 20S proteasome
Pter: Pterostilbene
PTPLAD1: 3-hydroxyacyl-CoA dehydratase 3
Ras: GTPase Ras
RNS: Reactive nitrogen species
ROS: Reactive oxygen species
SA: Senkyunolide A
SFN: Sulforaphane
SOD: Superoxide dismutase
SOD1: superoxide dismutase 1
SOM230: somatostatin analogs pasireotide
SQSTM1: sequestosome-1 protein
SR-B1: Scavenger receptor class B member 1
STIP1: stress induced phosphoprotein 1
TAC: Total antioxidant capability
TAK1: TGF beta-Activated Kinase 1
TGM2: transglutaminase 2
TLR4: toll like receptor 4
TNFα: tumor necrosis factor alpha
TRXR1: thioredoxin reductase 1
TUBB: tubulin beta class I
TUBB2A: tubulin beta 2A class IIa
TXN1: thioredoxin
UBB: Polyubiquitin-B
UB2R1: Ubiquitin-conjugating enzyme E2 R1
UGT: UDP glucuronosyl transferase
USP14: ubiquitin-specific peptidase 14
UV: ultraviolet
VCP: valosin-containing protein
